# Revising the stretch reflex threshold method to measure stretch hyperreflexia in cerebral palsy

**DOI:** 10.3389/fbioe.2022.897852

**Published:** 2022-11-23

**Authors:** Pedro Valadão, Lynn Bar-On, Francesco Cenni, Harri Piitulainen, Janne Avela, Taija Finni

**Affiliations:** ^1^ Neuromuscular Research Center, Faculty of Sport and Health Sciences, University of Jyväskylä, Jyväskylä, Finland; ^2^ Department of Rehabilitation Sciences, Ghent University, Gent, Belgium; ^3^ Department of Rehabilitation Medicine, Amsterdam Movement Sciences, Amsterdam University Medical Centers, Amsterdam, Netherlands; ^4^ Department of Neuroscience and Biomedical Engineering, Aalto University, Espoo, Finland; ^5^ Motion Analysis Laboratory, Children and Adolescents, Helsinki University Hospital, Helsinki, Finland

**Keywords:** hyperreflexia, neurophysiology, stretch reflex, electromyography, cerebral palsy

## Abstract

Hyper-resistance is an increased resistance to passive muscle stretch, a common feature in neurological disorders. Stretch hyperreflexia, an exaggerated stretch reflex response, is the neural velocity-dependent component of hyper-resistance, and has been quantitatively measured using the stretch reflex threshold (i.e., joint angle at the stretch reflex electromyographic onset). In this study, we introduce a correction in how the stretch reflex threshold is calculated, by accounting for the stretch reflex latency (i.e., time between the stretch reflex onset at the muscle spindles and its appearance in the electromyographic signal). Furthermore, we evaluated how this correction affects the stretch reflex threshold in children and young adults with spastic cerebral palsy. A motor-driven ankle dynamometer induced passive ankle dorsiflexions at four incremental velocities in 13 children with cerebral palsy (mean age: 13.5 years, eight males). The stretch reflex threshold for soleus and medial gastrocnemius muscles was calculated as 1) the joint angle corresponding to the stretch reflex electromyographic onset (i.e., original method); and as 2) the joint angle corresponding to the electromyographic onset minus the individual Hoffmann-reflex latency (i.e., latency corrected method). The group linear regression slopes between stretch velocity and stretch reflex threshold differed in both muscles between methods (*p* < 0.05). While the original stretch reflex threshold was velocity dependent in both muscles (*p* < 0.05), the latency correction rendered it velocity independent. Thus, the effects of latency correction on the stretch reflex threshold are substantial, especially at higher stretch velocities, and should be considered in future studies.

## Introduction

Hyper-resistance is defined as an increased resistance to passive muscle stretch, commonly reported in people with the upper motor neuron syndrome. Three main contributors to hyper-resistance have been identified: non-neural tissue properties, neural velocity-dependent stretch hyperreflexia and neural non-velocity dependent involuntary background activation (Gracies, 2005; Trompetto et al., 2014; van den Noort et al., 2017). Correctly assessing all components of hyper-resistance is crucial for treatment decision making and monitoring individual changes through life (e.g., effects of aging or a intervention; Tedroff et al., 2009; Tedroff et al., 2011). Stretch hyperreflexia is often characterized by the occurrence of the stretch reflex (SR) at abnormally lower stretch velocities and earlier joint angles (i.e., earlier in the stretch) compared to typically developing muscles. In clinical practice, manual stretch hyperreflexia assessment scales, such as the Modified Tardieu Scale (Boyd and Graham, 1999) have been widely used due to their ease of implementation without complex instrumentation requirements. The Modified Tardieu Scale dynamic range of motion test attempts to measure the joint angle at the SR torque onset (i.e., SR EMG onset plus electromechanical delay). The test consists of the examiner performing a fast passive stretch on the target muscle and measuring the angle of catch (i.e., angle at which muscle activity abruptly increases and stops the movement). Although simple to execute, this method is limited by the lack of stretch velocity and amplitude standardization, and inaccuracies related to subjectively measuring the angle of catch (van den Noort et al., 2009).

**FIGURE 1:**
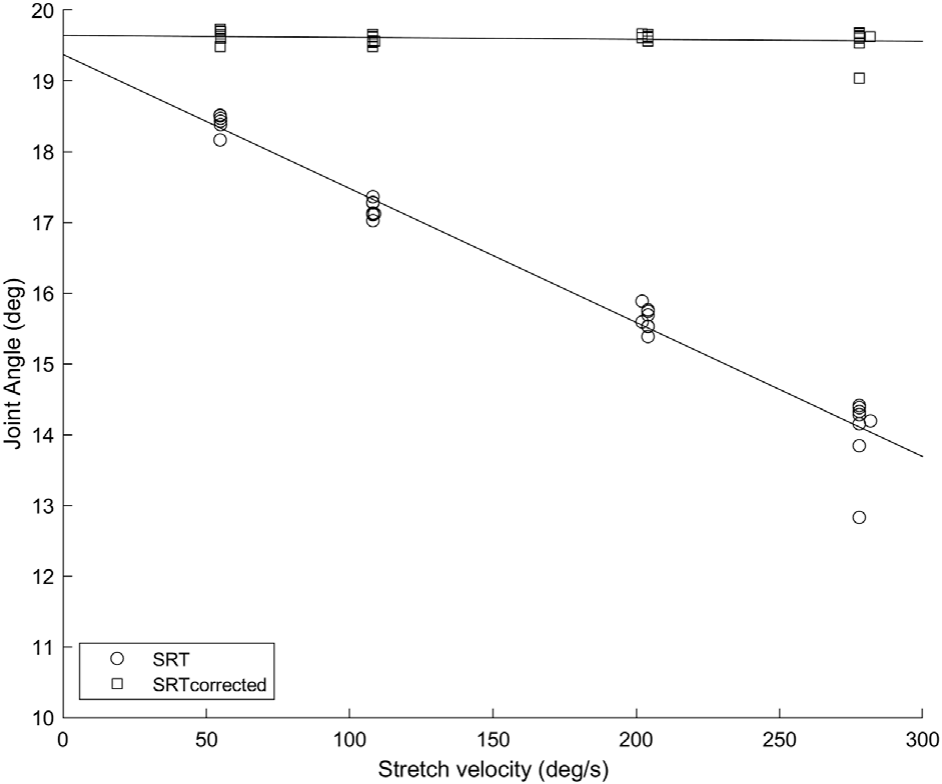
Soleus muscle SRTs (circles) and SRTs_corrected_ (squares) in repeated trials at four stretch velocities for a representative participant. The error caused by not considering the SR latency increases with increasing velocity, as the amount of angular displacement between the SR onset and the SR EMG onset is increasing.

To improve validity and reliability of stretch hyperreflexia assessments, quantitative methods utilizing recordings of joint kinematics and muscle electromyographic (EMG) activity have been developed, allowing more accurate assessment of the joint angle at the SR EMG onset, also termed stretch reflex threshold (SRT). The SRT can be measured at different stretch velocities, and it is generally assumed that higher stretch velocities will result in earlier onset joint angles (Levin and Feldman, 1994). Furthermore, the Tonic Stretch Reflex Threshold (TSRT) proposed by Levin and Feldman (Levin and Feldman, 1994) estimates a joint angle in which involuntary muscle activity would hypothetically start in the absence of joint movement. The TSRT is the y-intercept of the linear regression line through the SRTs with stretch velocity, thus representing the joint angle at zero velocity (Figure 1). Several studies have reported a moderate to high coefficient of determination (*R* ^2^) for the linear regression between the SRTs and stretch velocity (Calota et al., 2008; Blanchette et al., 2016; Germanotta et al., 2017; Frenkel-Toledo et al., 2021), which is vital for the validity of the TSRT (i.e., extrapolating the linear regression to zero velocity).

In the present study, we argue that the SRT and TSRT measures are influenced by a systematic error due to the lack of SR latency correction. SR latency is the duration between the SR being mechanically initiated at the muscle spindles (i.e., SR onset) to its appearance in the EMG signal (i.e., SR EMG onset). For a given SR latency, the difference between the joint angle at the SR onset and at the SR EMG onset (i.e., SRT) will have a positive linear relationship with the stretch velocity. For example, if we consider a SR latency of 30 ms and two stretches performed at 50°/s and 300°/s, the errors of using the angle at the SR EMG onset are 1.5° and 9° respectively, simply because the EMG onset is delayed by the ∼30 ms monosynaptic SR latency. Thus, without the SR latency correction, the SRT is progressively overestimated to later angles as velocity increases. While the study by Levin & Feldman (Levin and Feldman, 1994) acknowledged the SR latency problem in calculating the SRT and proposed subtracting 30 ms as a mean latency for the SR, later studies implementing the method did not make any correction. Since SR latency mainly depends on body dimensions associated with the axon pathway to the target muscle, subtracting an average value of 30 ms produces an unknown subject-specific systematic error. Therefore, the aim of the present study was twofold: 1) to correct the SRT calculation by considering the SR reflex latency and evaluate the effect of the correction on the linear relationship between SRT and stretch velocity; 2) to verify the validity of the TSRT method once the SRT is latency corrected. SR latency was estimated using the soleus (Sol) and medial gastrocnemius (MG) Hoffmann-reflex (H-reflex) latency. Thus, the SRT was computed as the joint angle at the SR EMG onset time (i.e., original method), and as the joint angle at SR EMG onset time minus the individual H-reflex latency time (i.e., latency correction method). We hypothesized that a significant change in the stretch velocity-SRT regression slope will occur due to the latency correction, as it will necessarily shift higher velocity SRT to earlier joint angles. Although the change in the regression slope is predictable, it is impossible to predict how the regression line *R* ^2^ will change, and thus the adequacy of the TSRT method. Furthermore, we hypothesized that the TSRT angle will not significantly change as the latency correction will have only a small effect on the lower velocity SRTs, and consequently will not change the y-intercept of the regression line considerably.

## Materials and methods

### Subjects

Thirteen children and young adults diagnosed with spastic cerebral palsy (CP), aged between 9 and 22 years participated in this study. None of the participants had lower limb surgery, serial casting, pharmacological treatments (except for oral medication) or had participated in a resistance-training program for the lower limbs in the past 6 months. All participants were able to stand with both heels touching the floor (i.e., ankle in anatomical position). Table 1 presents participant characteristics.

**TABLE 1:** Participant characteristics (*n* = 13).


Male/female	8/5
Mean (SD) age (years)	13.5 (4)
Mean (SD) height (cm)	159 (12)
Mean (SD) weight (Kg)	52 (16)
Level of involvement	Bilateral = 2/Unilateral = 11
GMFCS	I = 13

Data presented as mean (SD). GMFCS, gross motor function classification system.

### Study design

The present study is part of a larger nonconcurrent multiple-baseline research project called EXECP (Valadão et al., 2021), prospectively registered in the International Standard Randomized Controlled Trial (ISRCTN69044459). Data collected in Pre-tests 1 and 2 was utilized in this study.

### Experimental protocol

Detailed information about the testing procedures can be found in the research protocol (Valadão et al., 2021). EMG activity was recorded from Sol and MG muscles with self-adhesive electrodes (Blue Sensor N, interelectrode distance = 2 cm; Ambu, Ballerup, Denmark) following SENIAM (Hermens et al., 2000), and a ground electrode was placed on the tibia. EMG signals were amplified (gain 1,000) and high-pass filtered (10 Hz) by a preamplifier (NL824/NL820A; Digitimer, Welwyn Garden City, United Kingdom) and then band-pass filtered (20–195 Hz) off-line using Matlab software (v2020a, The Mathworks Inc, Natick, United States). The 20-Hz high-pass is suggested to offer the best compromise for optimizing the physiological informational content of surface EMG (De Luca et al., 2010), while the selected low-pass was chosen to eliminate high-frequency noise found in some EMG recordings (external laboratory noise). The H-reflex recruitment curve for Sol was elicited by percutaneous electrical stimulation of the tibial nerve at the popliteal fossa. H-reflex latency for both Sol and MG was determined by visual inspection as the duration between the electrical stimulus and the initial deflection of the H-reflex on the EMG signal. The H-reflex monosynaptic pathway is almost identical to the SR, except that the former is evoked by an electrical stimulus at the popliteal fossa, and the latter is generated by muscle spindles in the muscle. Subsequently, a motor-driven dynamometer (Neuromuscular Research Center, University of Jyväskylä, Finland) induced passive ankle dorsiflexions from 20° of plantarflexion to 0° at four angular velocities (55, 110, 210, and 291°/s). Ten stretches in each velocity were delivered in a pseudo-randomized and balanced order every 2.6–2.9 s. Participants were instructed to relax and wore noise blocking headphones. Moreover, trials with Sol or MG EMG root mean square computed over a 200 ms sliding window exceeding 5% of the maximal isometric plantarflexion test [see (Valadão et al., 2021)] in the 500 ms preceding the stretch were discarded. An EMG onset detection algorithm applying the approximated generalized likelihood principle (Staude and Wolf, 1999; Staude et al., 2001; Lee et al., 2007) was used to detect the SR EMG onset. Visual inspection was used to identify false positives and negatives generated by the algorithm, and manual onsets were set based on the criteria of the EMG signal reaching two standard deviations (SD) for a minimum of 100 ms. Since the stretch velocity is the independent variable and the SRT the dependent variable, the former has been assigned to the *x*-axis and the latter to the *y*-axis, which is the opposite of how this data has been presented in previous studies [e.g., (Calota et al., 2008; Blanchette et al., 2016)]. Thus, although the calculation of the *R* ^2^ values are the same between studies, the regression slopes are different, and the TSRT in the present study is the y-, rather than x-intercept.

### Data analysis

Data analysis was performed in Matlab (v2020a, The Mathworks Inc, Natick, United States). SRT was calculated in the original method as the joint angle at SR EMG onset time and with latency correction as joint angle at SR EMG onset time minus the individual H-reflex latency time for each muscle (SRT_corrected_). For example, if the Sol SR EMG onset happened 125 ms after the stretch onset and the participant’s Sol H-reflex latency is 25 ms, SRT is the joint angle 125 ms after the stretch onset, whereas SRT_corrected_ is the joint angle 100 ms after the stretch onset. The median SRT and SRT_corrected_ values for each subject at each stretch velocity were calculated for statistical analysis. TSRT and TSRT_corrected_ were calculated as the y-intercept of the regression lines between stretch velocity and SRT or SRT_corrected_, respectively.

### Statistical analysis

Data normality and equality of variances was tested with Shapiro-Wilk and Levene’s tests, respectively. The two-sided paired *t*-Test and the non-parametric analog Wilcoxon signed rank test were used to test differences between variables with and without latency correction. The Friedman test with the Bonferroni post hoc test was used to check the effect of stretch velocity on SRT and SRT_corrected_. All statistical analyses were performed in Matlab. Effect size between group means was calculated using Hedge´s g. Significance level was set at *p* < 0.05.

## Results

Results are presented as mean ± standard deviation for normally distributed data and median (interquartile range) otherwise. Figure 1 depicts an example of how the SRT data was used to calculate TSRT in the original method and with SR latency correction (TSRT_corrected_). Only participants with SRTs quantified in all four velocities were used for the statistical analysis (*n* = 12 for Sol, *n* = 11 for MG). Group Sol H-reflex latency was 28 ± 3 ms with a range of 23–33 ms and MG H-reflex latency 28 ± 4 ms, a range of 23–35 ms, which are in line with previous reports (Mazzocchio et al., 2001).

### Regression slope between SRT and stretch velocity

**FIGURE 2:**
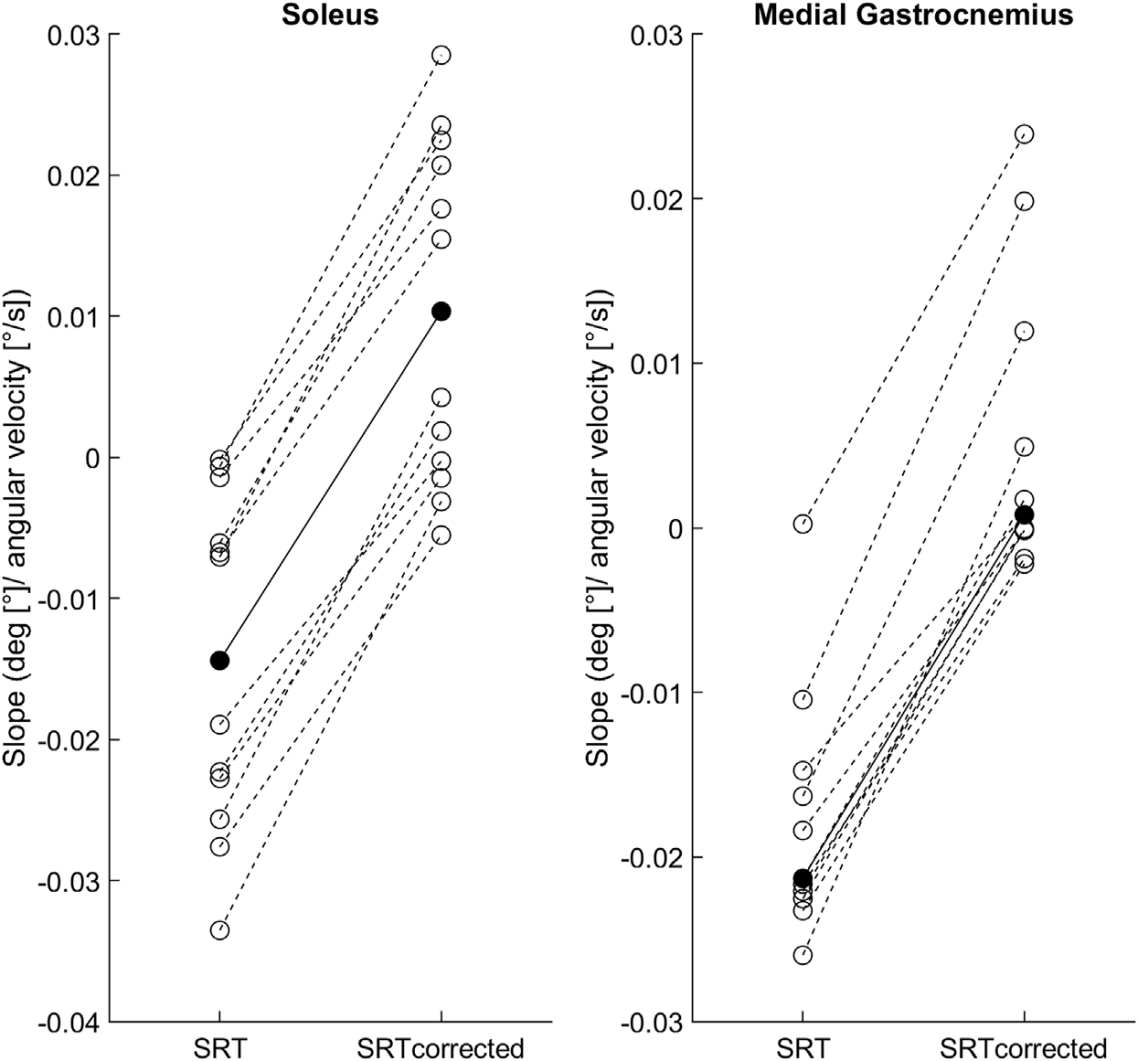
Individual SRT-velocity linear regression slopes (open circles) and soleus group mean/medial gastrocnemius median (filled circles) for both methods: original (SRT) and latency corrected (SRT_corrected_).

Figure 2 shows the individual and group mean or median slopes for the original and latency corrected methods. In Sol, the mean regression slope between the original (0.014 ± 0.012) and latency corrected (0.010 ± 0.012) methods were statistically different [t (11) = −19.3, *p* < 0.001, 95%CI = −0.03 to -0.02; hedge’s g = 2.0 (1.0–3.0)]. Similarly, in MG the median regression slope in the original method [−0.021 (0.01)] was statistically different from the latency corrected method [0.001 (0.01), *p* < 0.001].

### Effects of stretch velocity on SRT

In the original method, SRTs occurred at statistically different joint angles for both Sol (*p* = 0.008) and MG (*p* < 0.001). Bonferroni post-hoc analysis revealed that SRTs in the two slowest stretch velocities occurred significantly earlier than SRTs at the fastest (291°/s) velocity for both Sol (55°/s: *p* = 0.04; 110°/s: *p* = 0.009) and MG (55 and 110°/s: *p* < 0.001). With latency correction, no statistically significant differences across the stretch velocities were found for Sol (*p* = 0.552) or MG (*p* = 0.315). Table 2 shows the SRT results for the four stretch velocities.

**TABLE 2:** Effects of stretch velocity on Sol and MG SRT with and without latency correction.

Variables	Median (IQR) for stretch velocities			
	55°/s	110°/s	210°/s	291°/s
Sol SRT (°)	14 (13)*	16 (9)*	13 (5)	11 (4)
Sol SRT_corrected_ (°)	15 (13)	19 (9)	18 (5)	18 (3)
MG SRT (°)	18 (2)^†^	16 (1)^†^	14 (1)	11 (1)
MG SRT_corrected_ (°)	19 (2)	19 (1)	19 (1)	19 (1)

Sol, soleus; MG, medial gastrocnemius; SR, stretch reflex; SRT, stretch reflex threshold; IQR, interquartile range. Stretch velocities 55°/s and 110°/s are significantly different from 291°/s: *p < 0.05/^†^ *p* < 0.01.

### Coefficient of determination (R2)

**FIGURE 3:**
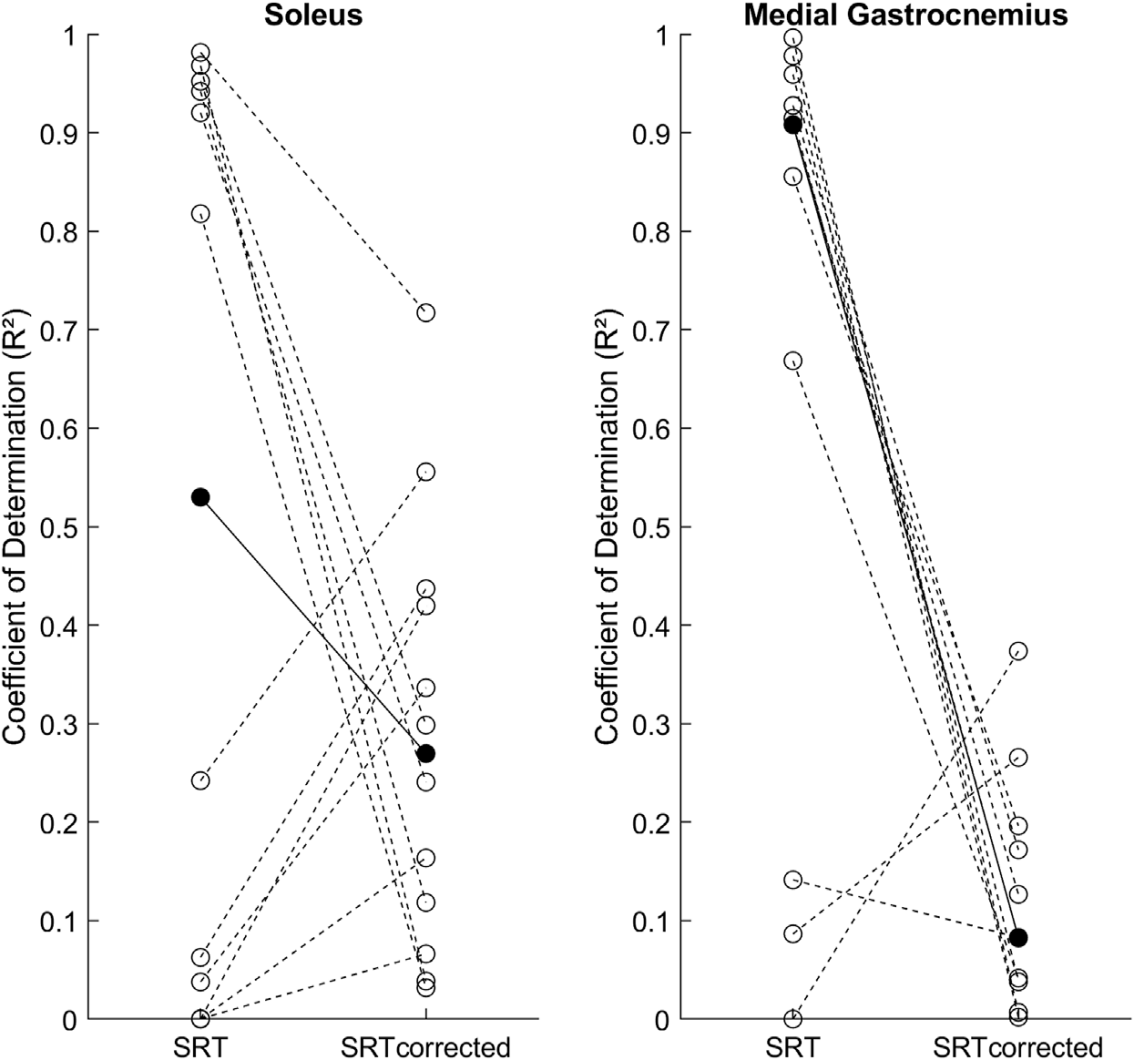
Individual SRT-velocity linear regression coefficients of determination (*R* ^2^, open circles) and group medians (filled circles) for both methods: original (SRT) and latency corrected (SRT_corrected_).

Figure 3 shows the individual *R* ^2^ results for the SRT-velocity linear regression and group medians for both methods. In Sol the *R* ^2^ between the original [0.53 (0.93)] and latency corrected [0.27 (0.34)] methods were not statistically different (*p* = 0.301). In MG, *R* ^2^ in the original method [0.91 (0.68)] was statistically higher than in the latency corrected method [0.08 (0.15), *p* = 0.01].

### TSRT

No statistically significant difference between Sol TSRT [16° (11)] and Sol TSRT_corrected_ [16° (11), *p* = 0.910] was found. Likewise, MG TSRT [18° (2)] and MG TSRT_corrected_ [19° (3), *p* = 0.102] were not statistically different.

## Discussion

The present work sought to verify the effects of the SR latency correction on the SRT and TSRT methods of stretch hyperreflexia assessment. The main findings were that latency correction significantly changed the SRT-velocity slopes and rendered the group-level SRT for both Sol and MG velocity independent. Thus, the lack of linear relationship between SRT and stretch velocity invalidates the use of a linear regression to find the TSRT.

### Regression slopes

To group individual SRT-velocity slopes, we defined a ‘near zero slope’ as having a modulus value smaller than 0.01, which would result in a maximum 2.5° difference between the slowest and fastest stretch velocities used in this study. Since within participant and velocity SRT median range was 1.5° (min–max range: 0.1–16°), a slope smaller than 0.01 would cause changes in SRT that are indistinguishable from the subject’s natural variability. At the individual level, with SR latency correction, six participants had near zero slopes in Sol (i.e., velocity independent) while the other six had positive slopes (i.e., earlier SRTs at higher velocities). In MG, nine participants had near zero slopes and two had positive slopes. Interestingly, all participants that showed velocity independency, had an early SRT within the first 2° of the stretch, whereas positive slopes were present in participants with SRTs later in the range of motion. These individual differences suggest that stretch velocity has a negligible effect on participants with early SRTs. A possible explanation may be that the muscle-tendon unit is already under tension and/or the IA arc is highly excitable (Nielsen et al., 2005).

The changes in the regression slopes caused by the SR latency correction towards positive values were expected since the correction shifts the SRTs of higher velocities to earlier angles. This means that when stretch velocity is increased, SRTs without the correction occurred progressively later in the range of motion, whereas the corrected SRTs occurred progressively earlier. Only the latter is an expected phenomenon of the velocity-dependent nature of hyperreflexia, and is also expected due to the viscoelastic behavior of the muscle-tendon unit [i.e., increased stretch resistance at higher velocities; (Taylor et al., 1990; Wu et al., 2010)].

### Coefficient of determination

Although the regression slope changes with SR latency correction were unidirectional (i.e., towards positive slope values), its effect on *R* ^2^ was bidirectional among our participants: 1) negative slopes shifting towards near zero slopes reduced *R* ^2^ (50% of participants in Sol and 82% in MG); 2) near zero slopes shifting towards positive slopes increased *R* ^2^ (50% of participants in Sol and 18% in MG). This explains why the statistically significant effect of SR-latency correction on *R* ^2^ was observed only in MG. The lower SRT variability in MG was probably due to the extended knee testing position, which placed the biarticular MG under considerably more tension than Sol. Overall, the low median latency corrected *R* ^2^ values for both muscles (Sol = 0.27, MG = 0.08) and high variability among participants, argues against the utilization of the linear regression to calculate the TSRT, at least in our sample. Nevertheless, previous studies have reported positive slopes without the SR latency correction for the same muscles that we studied (Blanchette et al. 2016 ; Germanotta et al. 2017). These studies would have had steeper slopes with the latency correction, and if a high *R* ^2^ was found, the TSRT method would be justified. Even though latency correction increased the slopes significantly in the current study, as expected, no differences in TSRT (i.e., y-intercept) were found between methods in both examined muscles. Since latency correction had a minimal effect on the SRTs at slow velocities (e.g., 28 ms * 55°/s = 1.5° correction), even considerable changes in the regression slope had small effect on the TSRT.

### Methodological remarks

Several important aspects of this study require further clarification: 1) a powerful motor-driven ankle-joint movement actuator was used to induce the stretches, whereas most of the aforementioned studies applied manual stretches. It took only 20–40 ms to achieve the target velocity in our actuator, which seems unlikely in manual stretches or even in the mechatronic device utilized by Germanotta et al. (2017), which had a maximum torque output of 7.1 Nm. Thus, it is possible that although mean joint velocities are comparable between studies, the joint acceleration profiles were very different (Sloot et al., 2021). Notably, during clinically applied manual tests such as the Modified Tardieu Scale, the stretch velocity is unknown making it impossible to perform the latency correction to the catch angle; 2) the stretch range of motion in the present study was shorter than other studies assessing the same joint (Blanchette et al., 2016; Germanotta et al., 2017). This was due to the extended knee testing position and the use of a motor driven dynamometer with end stop limits set for safety reasons. Fortunately, even in the slowest stretch velocity consistent SRs were evoked within this range of motion; 3) the H-reflex bypasses the muscle spindle and is evoked at the popliteal fossa, thus a small systematic error underestimating the SR latency by a few milliseconds is unavoidable, causing a small error in the SRT calculation at high stretch velocities. Nevertheless, the distance from the SR onset location in the tested muscles to the popliteal fossa could not be more than 15% of the entire IA arc pathway, thus for the current dataset it would represent a maximum of 4.2 ms (i.e., 15% of the mean latency) or an error of 1.2° in the fastest stretch velocity, still inferior to the within-subject and velocity SRT variability. Since using electrical nerve stimulation to assess SR latency is not feasible in most clinical setups, it would be very helpful to create easy measures using for example height and limb length that could estimate the SR latency of different muscles. Furthermore, since there is already considerable amount of data published on the subject, an effort to re-analyze it correcting for reflex latency would be of immense help for the scientific community; 4) the chosen EMG onset method performed very well in the fast stretches since the signal-to-noise ratio was very high. However, in the two slowest stretch velocities, many false positives and negatives were identified by visual inspection, and manual onset correction was extremely time-consuming. All onset corrections were logged, and the information will be available at the project’s repository. The lower SRT variability and better automatic EMG onset detection at higher stretch velocities strongly suggests designing SRT testing protocols with higher minimum stretch velocities.

The present study demonstrated that it is vital to consider SR latency when assessing the SRT and consequently the TSRT. To the best of our knowledge, most if not all current research utilizing the SRT as a measure of hyperreflexia has incurred in this error, thus a careful re-examination of data is important to update our understanding of this promising assessment method. Future research should assess the SRT and concomitantly measure muscle fascicle velocity (e.g., using ultrasonography) and the joint angular acceleration profile of the stretch. This information would allow better comparison between studies and perhaps elucidate why some participants exhibit velocity dependent SRT while others do not.

## Data Availability

Analysis scripts are available at https://github.com/Pedro-Valadao/EXECP_Neuromechanics. Raw data will be available upon request once EXECP’s sub-project I-SENS finishes data acquisition since it is not currently possible to anonymize the data.

## Abbreviations

CP, cerebral palsy; EMG, electromyography; H-reflex, Hoffmann-reflex; IQR, interquartile range; MG, medial gastrocnemius muscle; R2, coefficient of determination; SD, standard deviation; Sol, soleus muscle; SR, stretch reflex; SRT, stretch reflex threshold; TSRT, tonic stretch reflex threshold.
